# Correlation Between Core Stability and Plantar Pressure Distribution During Double-Leg Stance, Single-Leg Stance, and Squat Positions in Healthy Male Athletes

**DOI:** 10.3390/medicina61071188

**Published:** 2025-06-30

**Authors:** Reem Abdullah Babkair, Shibili Nuhmani, Turki Abualait, Qassim Muaidi

**Affiliations:** Department of Physical Therapy, College of Applied Medical Sciences, Imam Abdulrahman Bin Faisal University, P.O. Box 2435, Dammam 31451, Saudi Arabia; babkairra@gmail.com (R.A.B.); tsabualait@iau.edu.sa (T.A.);

**Keywords:** postural balance, weight bearing, foot, biomechanics, abdominal muscles, back muscles

## Abstract

*Background*: Core stability is a cornerstone of optimum athletic performance, and its reduction is a risk factor for athletic injuries. Evidence has shown that core impairments can alter lower-limb mechanics through the kinetic chains. Additionally, plantar pressure can be influenced by proximal conditions, such as core muscle fatigue. Therefore, this study aimed to investigate the correlation between core endurance and plantar pressure distribution (PPD) during double-leg stance, single-leg stance, and single-leg squat positions in healthy male athletes. *Methods*: A total of 21 healthy male recreational athletes between 19 and 26 years of age volunteered to participate in this correlational study. The McGill core endurance test was used to measure the endurance of their core flexors, extensors, and lateral flexors. The participants’ PPD was evaluated using the Tekscan Mobile Mat pressure measurement system in three positions (double-leg stance, single-leg stance, and single-leg squat) for both the dominant and non-dominant feet. *Results*: There was a poor and insignificant correlation (*p* > 0.05) between the core flexors’, extensors’, and side flexors’ endurance and the peak and total PPD in all the tested positions for both the dominant and non-dominant feet. *Conclusions*: Core muscle endurance is neither a component that affects nor is affected by the PPD in this study population. Thus, the endurance of core flexors, extensors, and side flexors may not be considered in screening, examination, or intervention for the total and peak pressure during double-leg stance, single-leg stance, and single-leg squat positions for both the dominant and non-dominant feet in the study population. Further similar studies are warranted in various sports and during dynamic tasks to better understand the different dimensions of the studied relationship in athletes.

## 1. Introduction

The ‘core,’ or ‘lumbopelvic-hip complex,’ includes the pelvis, lumbar vertebrae, hip joints, and the muscles and tendons that influence their movement. Both active and passive components contribute to core stability, along with neural elements within the central nervous system. Core stability maintains structural integrity and limits displacements during static and dynamic conditions, providing proximal stability for optimal distal mobility [1]. Studies show that extremity movement is precipitated by core muscle activation, as observed through electromyography [2]. This is believed to occur via the kinetic chain concept, a fascial link that connects the core to the upper and lower limbs, forming a network across body parts. This connection is evident in studies linking core stability exercises with performance tests for the upper and lower extremities [3].

Core stability requires various components, including muscle strength, endurance, proprioception, and neuromuscular control, with a power element added during athletic activities. The integration of these constructs ensures optimal core stability and function at the extremities. In sports, the focus on a specific core stability component varies by the sport’s needs. Among those components, core endurance is commonly tested, defined as the ability to maintain core position or perform low-load tasks repeatedly without fatigue [4,5,6]. It is vital for muscle coordination, spinal stabilisation, and sustaining athletic performance [7].

Many studies on sports like running, hockey, tennis, soccer, and basketball discussed the link between core endurance, athletic performance, and injuries [6,8]. Core endurance is related to athletic performance metrics and exercises, including single-leg squats, vertical jumps, push-ups [9], the 20-metre sprint [10], Yo-Yo tests [9], Y-balance tests [11], and medicine ball throws [12]. Additionally, core endurance is positively correlated with athletic injuries such as running-related injuries in rugby players, low back pain in equestrian athletes, and knee and ankle injuries in soccer players [13,14,15].

Plantar pressure distribution (PPD), or pedobarography, examines pressure on the foot’s plantar surface. Measuring PPD aids in analysing balance and foot function, designing custom shoes, and preventing injuries. Increased PPD is a key risk factor for foot and lower-limb injuries like stress fractures and ankle sprains, while even distribution contributes to better athletic performance. It is worth noting that PPD can be affected by leg muscle fatigue [16,17,18] and, more proximally, by core muscle fatigue [19].

Considering that core stability (endurance component) can have an influence, distally, on the mechanics of the lower extremities via the fascial link and that PPD is affected by proximal structures such as core fatigue, to the best of the author’s knowledge, the relationship between core endurance and PPD has not yet been established. Therefore, our study aimed to investigate the relationship between PPD and core endurance in static positions, including double-leg stance, single-leg stance, and single-leg squat, in healthy male athletes. The study findings may provide valuable information for use in screening methods, injury prevention programs, treatment protocols, and other applications to save costs and time lost due to sports injuries.

## 2. Materials and Methods

Ethical approval for the study was obtained from the Institutional Review Board Committee at Imam Abdulrahman bin Faisal University in Dammam, Kingdom of Saudi Arabia (approval number: IRB-PGS-2022-03-086), on 20 February 2022.

### 2.1. Participants

This cross-sectional study was conducted at the Physical Therapy Laboratory, Physical Therapy Department, College of Applied Medical Sciences, Imam Abdulrahman bin Faisal University, Dammam. A sample size of 21 participants was determined using https://www.ai-therapy.com/psychology-statistics/sample-size-calculator, (accessed on 10 January 2022) powered to detect the relationship between core endurance and PPD. Based on previous research linking arch height and PPD in a normal population [20], the current study assumed a correlation coefficient of 0.7, a significance level (*p*) of 0.05, and an expected power (β) of 0.8. Twenty-one healthy male recreational athletes aged 21.3 ± 1.65 years, weighing 71.65 ± 7.84 kg, with an average height of 171.33 ± 6.05 cm and a BMI of 24.41 ± 2.65, participated in the study. Recreational athletes were defined as individuals who participate regularly in sporting events one to three times per week [21]. Exclusion criteria included recent limb/trunk injuries, back pain requiring medical attention, fractures, surgeries, lower-limb deep vein thrombosis, neurological deficits, systemic diseases, chronic illnesses, concussions, or biomechanical deformities. Additionally, athletes on medications that could influence testing, those reporting pain, and smokers were also excluded. The information mentioned earlier was collected verbally, as reported by participants, and through inspection and palpation performed by the primary researcher (RB). Eligible athletes refrained from strenuous activities for 24 h before testing. The study procedure was explained, and informed consent was obtained from all the participants. Participants had the right to withdraw from the study at any time without consequences.

### 2.2. Procedure

**Figure 1:**
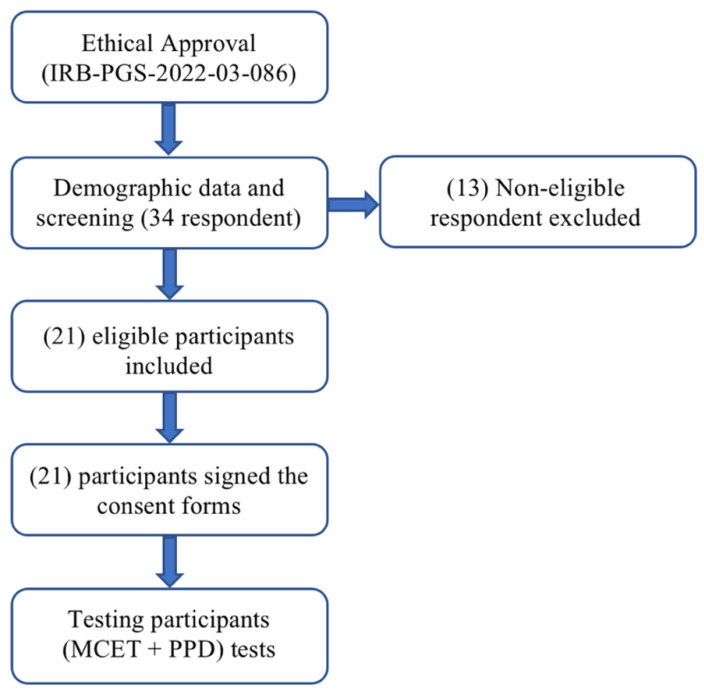
The flow chart of the study procedure.

The study protocol began with participant screening and consent, followed by testing procedures. The timeline of the study procedure is illustrated in Figure 1. The dominant lower limb was identified based on participants’ preferred limb for three tasks: kicking an imaginary ball, drawing the numeral ‘8’ on the floor, and extinguishing an imaginary fire [22]. Participants were not informed about the purposes of the tasks or which limb to use beforehand. The limb used instinctively in two or more tasks was deemed the dominant limb [22]. The testing environment, lap temperature, and testing timing were standardised among the study participants. It was reported that an improvement in endurance training performance was observed during daytime [23]. Therefore, collecting data was standardised among all participants during the duty hours from 10:00 am to 3:00 pm, assuming that the current study participants are (morning chronotype) since they are all either university students or daytime employees. Moreover, the lab temperature was centrally preset to 24 °C to ensure comfort for all participants. Racinais et al. (2005) concluded that muscle contractility is improved when daytime and warm climate factors are combined [24].

### 2.3. Overview of Outcome Measures

The current study utilised clinical tests for core endurance and a device for PPD assessment. Core endurance was evaluated using the McGill Core Endurance Test (MCET), a static isometric endurance measure that includes four tests for core flexors, extensors, and side flexors. PPD was measured with the Tekscan MobileMat pressure measurement system (Tekscan, Inc., South Boston, MA, USA) in three positions: double-leg stance, single-leg stance, and single-leg squat. To minimise variability, all tests of core endurance and PPD were administered by the primary researcher (R.B). Tests were performed randomly to avoid learning and order effects. For recovery, a 5 min rest was allowed between core endurance tests [4]. A coin toss determined which limb to test first (right or left) before PPD testing during the single-leg stance and squat. Each testing procedure lasted about 45 min per participant. The timeline of the outcome measures is shown in Table 1.

### 2.4. Core Endurance Testing

MCET assessed core endurance through four tests: trunk flexor, trunk extensor, and right and left lateral plank. All tests were performed on a floor mat, except the trunk extensor, which was on a plinth. Participants were instructed to maintain specific positions for as long as possible. They received no encouragement or timing information [27]. Testing time was recorded in seconds.

#### 2.4.1. Core Flexor Endurance Test

Participants were seated on a sports mat with their backs flat against a wooden wedge positioned at 60°, hands across their chest, and knees at 90° flexion. The researcher measured knee joint angles using a universal goniometer. The axis of the goniometer was aligned with the knee joint, with the top arm placed on the lateral thigh towards the greater trochanter and the lower arm on the lateral leg towards the lateral malleolus [28]. The same method was used for the other knee. When both knee angles were at 90° flexion, the assistant moved the wedge 10 cm away from the participant, guided by a 10 cm line pre-drawn on the floor. The investigator then said ‘Start’ to record the time. The time was stopped when the participant’s trunk moved from the starting position [27,29]. This test has been reported to have an excellent test–retest reliability, with ICC scores ranging from 0.95 to 0.98 [4].

#### 2.4.2. Core Extensor Endurance Test

Participants took the test in a prone position, with all body parts above the anterior superior iliac spine positioned off the plinth and their ankles placed on a small pillow. Three Velcro straps stabilised their lower bodies: one above the ankles, one above the knees, and one above the gluteal fold. They rested their palms on a stand placed on the floor at shoulder level vertically while an assistant stabilised their legs. When ready, participants folded their arms across their chests. When their trunk was parallel to the floor, the researcher said ‘start’ and began recording time. The recording stopped upon visual detection of trunk deviation. This test exhibits good reliability, with ICC scores ≥ 0.77 [30].

#### 2.4.3. Left and Right Lateral Plank Tests

In a side-lying position on the mat, participants placed their top foot over the lower and propped their body up using their elbows and feet. The elbow was at 90° flexion, aligned with the shoulder, and the top arm rested on the torso [31]. Participants were instructed to keep their trunk, pelvis, and legs straight, with both knees extended. When the correct side plank position was achieved, the researcher said ‘start’ and began recording time. If the researcher visually noticed any trunk deviation from alignment, they said ‘stop’ and ceased recording [27]. These tests have shown excellent intra-rater reliability, with ICC scores of ≥0.97 [4].

### 2.5. Plantar Pressure Distribution Testing

The Tekscan Mobile Mat pressure measurement system (Tekscan, Inc., Boston, MA, USA) with FootMat Clinical Software version 7.00-65 measured PPD. This reliable [32], user-friendly device, used in previous studies [33,34], features a 2.5 × 2.5-foot mat that senses PPD through paper-thin embedded sensors and connects to a computer via USB.

The step calibration method was employed since the activities to be performed (i.e., double-leg stance, single-leg stance, and single-leg squat) require participants to maintain static positions. To set up (step) calibration, participants started with both feet off the FootMat. The researcher entered the participants’ weights and clicked the start button on the computer screen, after which a timer appeared in the calibration window. After a few seconds, the system required participants to step with one foot (left or right) on the FootMat and keep the assumed position for five to ten seconds. Once the calibration was successful, real data collection started. A “Step calibration” was performed for each participant individually before starting real data collection as recommended by the Tekscan user manual.

#### Collecting Plantar Pressure Data

**Figure 2:**
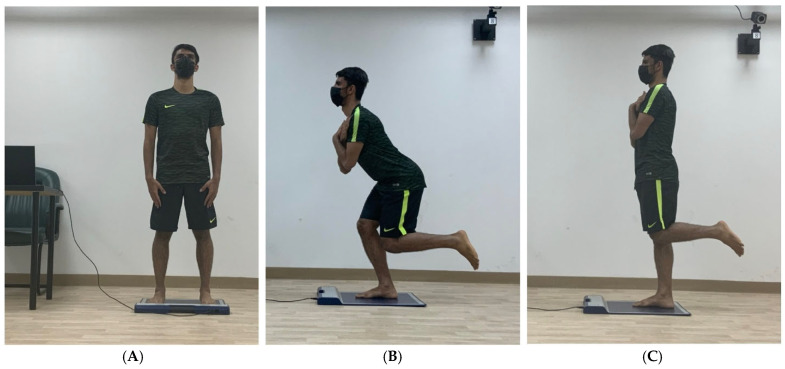
Measurement of plantar pressure distribution, (**A**). in double-leg stance position, (**B**). single-leg stance position, and (**C**). single-leg squat position.

All PPD tests were conducted barefoot to ensure sensor contact and standardisation. Each participant completed two official trials for PPD measurement in double-leg and single-leg stances (left and right) and single-leg squats (left and right) to reduce errors. The official two trials were preceded by two practice trials in each tested position [26]. The researcher averaged participants’ scores from the official trials for analysis. Each trial lasted 6 s [33]. To gather PPD data in the double-leg stance, participants simply stood on the testing mat with feet shoulder-width apart and arms at their sides, looking forward (Figure 2).

To collect PPD data in a single-leg stance, each participant stepped onto the FootMat with one foot. The non-supporting limb was at 90° knee flexion, aligned but not touching the supporting limb. The researcher measured the knee joint angle using a universal goniometer, placing the axis on the knee joint. The top arm was positioned on the lateral thigh, pointing to the greater trochanter, while the lower arm pointed to the lateral malleolus on the lateral leg [28]. Participants folded their arms against their chest and looked forward (Figure 2). They maintained this position for 6 s as PPD data were recorded [28].

PPD data were collected during a single-leg squat, as described by Gwynne [35], but the degree of squatting on a single limb was chosen after [36]. Participants stepped onto the FootMat with the tested leg while the non-supporting leg was at 90° knee flexion, aligned but not touching. A goniometer measured the angle of the non-supporting knee, similar to the PPD measurement in single-leg stance. Participants folded their arms to eliminate the swing and looked forward. They squatted on the tested leg to about 30° knee flexion, maintaining balance (Figure 2). The researcher also measured the supporting leg’s knee angle to confirm the squatting degree. Participants held the position for 6 s while PPD data were recorded. Participants were asked to maintain a neutral foot position during the squat.

### 2.6. Data Analysis

Statistical analysis was carried out using the SPSS version 27 (IBM Corp., Armonk, NY, USA) software. The Shapiro–Wilk test was used to check the normality of the data. Pearson’s correlation test was used to correlate between normally distributed data, while with non-normally distributed data, Spearman’s correlation test was used. The significance level was set at <0.05. The correlation strength was interpreted as follows: +/− (>0.8), +/− (0.7–0.8), +/− (0.5–0.6), +/− (0.3–0.4), and +/− (0.0–0.2) represent almost perfect, strong, moderate, fair, and poor correlations, respectively [37].

## 3. Results

The means and SDs of the normally distributed data are provided in Table 2, and the medians and 25–75 percentiles of the non-normally distributed data are provided in Table 3. The correlations between the study variables were made according to the normality of the data. A total of 48 correlations between the participants’ MCET test and PPD data were measured. All the correlations were non-significant, with strengths ranging from poor to fair (Table 4).

### Additional Findings

Table 5 shows weak and insignificant correlations between the PPD values of the dominant foot in the tested static positions, except for the significant, moderate, and positive relationship of the total and peak PPD of the dominant foot in the double-leg stance with the peak PPD in the single-leg stance (r = 0.527, *p* = 0.014; r = 0.570, *p* = 0.007). As for the non-dominant foot, Table 6 also shows weak and insignificant correlations between its PPD values in the tested static positions.

## 4. Discussion

This study investigates the link between core muscle endurance and PPD in three static positions among healthy male athletes. To the authors’ knowledge, it is the first to correlate these factors in both athletic and non-athletic populations. The chosen static positions are key to establishing basic knowledge in sports performance, and injury prevention, paving the way for advanced sport-specific research. The chosen static positions are sport-specific [38,39,40] and used in practice and performance testing [41,42]. Single-leg stances and squats are linked to various athletic injuries [43,44,45]. Recreational athletes aged 18 to late 30s were selected for this study, as they are typically in peak health, physically and mentally mature, and self-directed [46]. They excel physiologically in muscle strength, cardiac function, sensory abilities, and reaction time, placing them at the peak of athletic performance and profession [46].

This study finds no correlation between MCET test scores and PPD values (total and peak pressure) in static positions (double-leg stance, single-leg stance, and single-leg squat) among healthy male athletes. Previous studies explored core and leg muscle fatigue’s effect on PPD during dynamic activities [16,17,18,19]. For instance, one study with 46 novice runners found a positive relationship between core muscle fatigue and PPD [19]. Muscular fatigue leads to tiredness and reduced performance. Askari and Esmaeili [19] findings contradict this study’s lack of correlation between core muscle endurance and PPD, likely due to different testing positions: cyclic dynamic in their research versus static in the present study. Thus, core endurance seems more related to PPD in dynamic than static activities [19].

Weist, Eils, and Rosenbaum [16] studied the effect of leg muscle fatigue on PPD during running in 30 experienced runners and found that leg muscle fatigue increased PPD under the medial aspect of the midfoot and the second and third metatarsal heads. Lung, Liau, Peters, He, Townsend, and Jan [17] observed changes in PPD induced by tibialis anterior muscle fatigue during walking and running, and found an increase in peak PPD under the big toe and first metatarsal head. Zhao [18] investigated the effect of leg muscle fatigue on PPD during walking in college students. After fatigue, plantar loading was reduced under the medial arch, distal metatarsals, rear heel, and cuboid bone. Despite the conflicting results of these studies, it is evident that there is a relationship between leg muscle fatigue and PPD during dynamic activities, which negates our findings. Based on the idea that fatigue depletes endurance, it may be concluded that leg muscle endurance also has a correlation with PPD during dynamic activities.

On the other hand, Vie, Gomez, Brerro-Saby, Weber, and Jammes [47] investigated the effect of leg muscle fatigue on the measurement of plantar surface area/cm^2^ during the double-leg standing position (static). The researchers found that leg muscle (tibialis anterior) fatigue increased the mean pressure and the centre of the pressure surface area/cm^2^ in 12 healthy male subjects [47]. Again, if fatigue is considered the opposite of endurance, leg muscle endurance may be related to PPD measured in static positions, while in dynamic activities, both core and leg endurance may have a correlation with PPD. Accordingly, a question may be raised as to whether a positive correlation might have been found in the current study if leg muscle endurance were measured and correlated with PPD. Seeking to answer this question may pave the way for future research.

Finally, Lee, Wang, Lee, Yu, Kim, Kim, and heon Hong [48] conducted a study on 21 healthy male and female adults to compare the influences of progressive ankle- and core-muscle-strengthening exercises on static and dynamic balance. The exercises were performed for four weeks. Static balance was measured with stability and weight distribution indices using a Tetrax^®^ device. The dynamic balance of both legs was evaluated using the Y-balance test. It was concluded that both static and dynamic balance abilities were positively affected by the progressive theraband exercises. Thus, core-muscle-strengthening exercises were reported to be more effective with dynamic balance, while ankle-muscle-strengthening exercises were more effective with static balance. Considering that the weight distribution in PPD measurement is directly related to balance, PPD can be used as a measurement of balance and postural sway [49]. The Findings of Lee, Wang, Lee, Yu, Kim, Kim, and heon Hong [48] can perhaps support the proposition that PPD is more related to leg endurance in static positions and to core endurance in dynamic activities.

### 4.1. Additional Findings

The findings in Table 5 and Table 6 reveal weak, insignificant correlations between PPD measurements across almost all tested positions in both dominant and non-dominant feet. This indicates that PPD measurements in these positions are independent. This novel discovery fills a significant gap in current knowledge. While some studies have examined PPD values during dynamic sports activities [50] or in specific static positions like the single-leg squat [51], none have compared PPD differences across various static positions. The minimal correlation may stem from differing muscle activation in each position, as lower-limb activation varies between double-leg stance [52], single-leg stance [53], and single-leg squat [51]. Thus, the muscle activity variance in these static positions could influence PPD parameters, making the lack of correlation understandable. Nevertheless, further research is necessary to clarify the reasons behind the lack of correlation between PPD in the tested positions.

The previous finding has one exception. Table 5 shows a significant, moderate, positive correlation between the total and peak PPD of the dominant foot in a double-leg stance and peak PPD in a single-leg stance. This novel finding fills a knowledge gap, appearing only in the dominant foot during single-leg stance (not in the non-dominant foot). This may result from the lack of comparability of PPD between feet [54]. Static peak PPD significantly differs between dominant and non-dominant feet, with higher static forces linked to body weight on the dominant side [54]. Further research is needed to identify the reasons for this relationship.

### 4.2. Clinical Implications

The study findings suggest no correlation between most PPD values for the dominant and non-dominant feet, except for the total and peak PPD in the double-leg stance, which correlated with the peak PPD of the dominant foot in the single-leg stance. Thus, PPD values are largely independent. When PPD measurement is needed to customise insoles to meet sports demands, it may be important to conduct it in sport-specific positions. Further investigation into these findings is recommended.

### 4.3. Study Limitations

Recruiting only male recreational athletes limited the generalisability of findings to other populations. However, it improved internal validity within the tested group. Another limitation was including individuals from various sports, which reduced sample homogeneity and internal validity but enhanced external validity. Additionally, the MCET test assessed only part of the lower core musculature, boosting internal validity while lowering external validity. Moreover, prolonged sitting weakens back extensor muscles and endurance. This variable was not addressed. Participants’ internal motivation during data collection, which may have affected core muscle endurance values, was not assessed. However, the effect may be minimised since no external motivational talk was given before testing. Also, foot type is a risk factor for changes in PPD values [55]. Research shows that core lateral flexor endurance is lower in university students with flat feet than healthy peers, yet this was only visually inspected here.

The quadriceps angle influences PPD values but was not measured in this study. The lack of control over participants’ fat profiles, like waist circumference and calf girth, is another limitation. While BMI classifies weight status, it does not measure body fat or muscle mass. Despite these limitations, the study offers valuable insights into core endurance and PPD in healthy male recreational athletes, providing guidance for athletes, therapists, and sports organisations to save time and costs.

### 4.4. Recommendations for Future Studies

Future research may confirm the results of the current study and consider different populations, such as females, the elderly, and individuals with special needs. It may also consider the psychological, nutritional, and lifestyle factors of participants, along with different core stability constructs (strength, power, proprioception). Additionally, researching the correlation of core and leg muscle endurance with PPD in both static and dynamic settings will clarify how endurance relates to PPD in sports, carrying important clinical implications.

## 5. Conclusions

This study concludes that trunk flexor, extensor, and side flexor endurance are not relevant for screening foot pressure during double-leg stance, single-leg stance, and single-leg squat. Therefore, core endurance neither influences nor is influenced by PPD measurements in this population.

## Figures and Tables

**Table 1:** The timeline of the outcome measure testing.

Outcome Measures	Testing Sequence
The sequence of the testing procedure was undertaken randomly for both McGill core endurance and PPD tests (participants chose from folded papers blindly) to rule out that one test might affect the result of the other.	
McGill core endurance tests: - Flexors endurance test. - Extensors endurance test. - Left lateral plank test. - Right lateral plank test.	Explanation and demonstration of the testing position and procedure was provided to each participant. - Each participant was allowed to practice the testing position for no longer than 5 s to avoid fatigue. - One official trial was completed only to avoid fatigue [25]. - Once participants assumed the desired position, the official trial started with the word “Start” and ended with “Stop” upon the trunk deviation from the midline noticed. - The holding time was recorded in seconds. - Participants were allowed to rest for five minutes between the testing trials to eliminate the effect of fatigue on the next test’s performance [4].
Plantar pressure distribution measurement: Data were recorded during the following static positions: - Double-leg stance. - Single-leg stance. - single-leg squat.	For all the testing positions: - Explanation and demonstration of the testing position and procedure was provided to each participant. - Two practicing trials before the formal ones were allowed. - Two formal trials were conducted [26]. - Formal trial recording started (after assuming the testing position) with the word “Start” and ended with “Stop”. - Each formal trial lasted six seconds. - The average of the two formal trials was taken in data analysis. For PPD testing in single-leg stance and squat positions: - The selection of which foot to test first (dominant or non-dominant) was made randomly after a coin flip. - Before recording, the researcher measured (using a Goniometer) the non-supporting leg’s 90° knee flexion in the single-leg stance and single-leg squat positions. Besides measuring the supporting leg’s 30° knee flexion in the single-leg squat position.

PPD: Plantar pressure distribution.

**Table 2:** Normally distributed variables’ mean and standard deviation.

Normally Distributed Variables	Mean ± Standard Deviation
McGill core endurance test	
Extension test (s)	34.32 ± 13.88
Left lateral plank test (s)	30.76 ± 14.50
Bilateral stance average values of plantar pressure distribution	
DPPA	22.81 ± 10.32
NDPP	25.50 ± 9.97
DPA	16.61 ± 6.59
NDPA	18.40 ± 6.00
Single-leg stance average values of plantar pressure distribution	
DPPA	47.09 ± 7.15
NDPPA	50.42 ± 10.34
DPA	32.90 ± 6.34
NDPA	38.04 ± 7.51
Single-leg squat average values of plantar pressure distribution	
DPPA	53.85 ± 9.58
NDPPA	52.61 ± 8.52
DPA	35.52 ± 7.19
NDPA	34.57 ± 5.32

DPPA, dominant leg peak plantar pressure average; NDPPA, non-dominant leg peak plantar pressure average; DPA, dominant leg total plantar pressure average; NDPA, non-dominant leg total plantar pressure average.

**Table 3:** Non-normally distributed variables’ median and 25–75 percentiles.

Non-Normally Distributed Variables	Median (25–75 Percentiles)
McGill core endurance test	
Flexion test (s)	74.38 (47.78–109.13)
Right lateral plank test (s)	24.63 (15.76–35.02)

**Table 4:** Correlations of the tested variables.

Plantar Pressure Data	McGill Core Endurance Tests			
	Flexion Endurance Test	Extension Endurance Test	Right Lateral Plank Test	Left Lateral Plank Test
Double-leg stance position				
DPPA	rho = −0.117 *p* = 0.614	r = 0.148 *p* = 0.523	rho = 0.068 *p* = 0.769	r = 0.030 *p* = 0.896
NDPPA	rho = 0.114 *p* = 0.622	r = 0.159 *p* = 0.490	rho = 0.112 *p* = 0.630	r = 0.034 *p* = 0.883
DPA	rho = −0.149 *p* = 0.520	r = 0.125 *p* = 0.590	rho = 0.109 *p* = 0.640	r = 0.061 *p* = 0.794
NDPA	rho = 0.129 *p* = 0.578	r = 0.215 *p* = 0.348	rho = 0.050 *p* = 0.829	r = 0.060 *p* = 0.796
Single-leg stance position				
DPPA	rho = −0.079 *p* = 0.732	r = −0.025 *p* = 0.915	rho = 0.120 *p* = 0.605	r = 0.175 *p* = 0.449
NDPPA	rho = −0.040 *p* = 0.865	r = −0.299 *p* = 0.188	rho = 0.136 *p* = 0.555	r = −0.104 *p* = 0.653
DPA	rho = −0.024 *p* = 0.918	r = −0.331 *p* = 0.142	rho = 0.112 *p* = 0.630	r = 0.011 *p* = 0.963
NDPA	rho = 0.025 *p* = 0.913	r = −0.297 *p* = 0.191	rho = 0.279 *p* = 0.220	r = 0.078 *p* = 0.737
Single-leg squat position				
DPPA	rho = 0.364 *p* = 0.105	r = 0.341 *p* = 0.131	rho = 0.068 *p* = 0.769	r = −0.051 *p* = 0.826
NDPPA	rho = 0.182 *p* = 0.430	r = 0.147 *p* = 0.526	rho = −0.178 *p* = 0.440	r = −0.146 *p* = 0.527
DPA	rho = 0.331 *p* = 0.143	r = 0.307 *p* = 0.176	rho = 0.003 *p* = 0.991	r = 0.111 *p* = 0.631
NDPA	rho = 0.337 *p* = 0.135	r = 0.257 *p* = 0.261	rho = −0.071 *p* = 0.760	r = 0.164 *p* = 0.476

DPPA, dominant leg peak plantar pressure average; NDPPA, non-dominant leg peak plantar pressure average; DPA, dominant leg total plantar pressure average; NDPA, non-dominant leg total plantar pressure average; r, Pearson’s correlation coefficient; rho, Spearman’s correlation coefficient; *p*, *p*-value =< 0.05.

**Table 5:** Correlations between plantar pressure measurements of the dominant leg.

	Double-Leg Stance DPPA	Double-Leg Stance DPA	Single-Leg Stance DPPA	Single-Leg Stance DPA	Single-Leg Squat DPPA	Single-Leg Squat DPA
Double-leg stance DPPA						
Double-leg stance DPA						
Single-leg stance DPPA	r = 0.570 *p* = 0.007 *	r = 0.527 *p* = 0.014 *				
Single-leg stance DPA	r = 0.335 *p* = 0.137	r = 0.320 *p* = 0.157				
Single-leg squat DPPA	r = 0.088 *p* = 0.706	r = 0.031 *p* = 0.894	r = 0.086 *p* = 0.711	r = −0.052 *p* = 0.823		
Single-leg squat DPA	r = 0.283 *p* = 0.213	r = 0.208 *p* = 0.367	r = 0.310 *p* = 0.172	r = 0.236 *p* = 0.302		

DPPA, dominant leg peak plantar pressure average; DPA, dominant leg total plantar pressure average; r, Pearson’s correlation coefficient; *p*, *p*-value =< 0.05; * Statistically significant at p < 0.05

**Table 6:** Correlations between plantar pressure measurements of the non-dominant leg.

	Double-Leg Stance NDPPA	Double-Leg Stance NDPA	Single-Leg Stance NDPPA	Single-Leg Stance NDPA	Single-Leg Squat NDPPA	Single-Leg Squat NDPA
Double-leg stance NDPPA						
Double-leg stance NDPA						
Single-leg stance NDPPA	r = 0.265 *p* = 0.245	r = 0.210 *p* = 0.362				
Single-leg stance NDPA	r = 0.110 *p* = 0.634	r = 0.108 *p* = 0.640				
Single-leg squat NDPPA	r = 0.091 *p* = 0.695	r = 0.051 *p* = 0.827	r = 0.247 *p* = 0.280	r = 0.210 *p* = 0.362		
Single-leg squat NDPA	r = 0.284 *p* = 0.212	r = 0.247 *p* = 0.281	r= 0.006 *p* = 0.978	r = −0.137 *p* = 0.554		

NDPPA, non-dominant leg peak plantar pressure average; NDPA, non-dominant leg total plantar pressure average; r, Pearson’s correlation coefficient; *p*, *p*-value =< 0.05.

## Data Availability

The data that support the findings of this study are available from the corresponding author upon request.
